# Exploration of (*R*)-[^11^C]YH168 as a PET tracer for imaging monoacylglycerol lipase in the brain: from mice to non-human primates

**DOI:** 10.1007/s00259-024-07013-0

**Published:** 2024-12-14

**Authors:** Yingfang He, MingQiang Zheng, Jiwei Gu, Lisa Reichert, Johannes Trimborn, Hui Zhang, Claudia Keller, Mallory Crosby, Ludovic Collin, Dominik Heer, Anto Pavlovic, Andreas Topp, Matthias Beat Wittwer, Uwe Grether, Luca Gobbi, Roger Schibli, Yiyun Huang, Linjing Mu

**Affiliations:** 1 Center for Radiopharmaceutical Sciences, Institute of Pharmaceutical Sciences, Department of Chemistry and Applied Biosciences, ETH Zurich, Zurich, CH-8093 Switzerland; 2 Yale PET Center, Department of Radiology and Biomedical Imaging, Yale University, New Haven, CT USA; 3 Pharma Research and Early Development, Roche Innovation Center Basel, F. Hoffmann-La Roche Ltd, Basel, CH-4070 Switzerland; 4 Institute of Radiation Medicine, Fudan University, Xietu Road 2094, Shanghai, 200032 China

**Keywords:** Monoacylglycerol lipase, Carbon-11, Neuroimaging, Drug development

## Abstract

**Purpose:**

The monoacylglycerol lipase (MAGL) plays a pivotal role in modulating the endocannabinoid system and is considered an attractive therapeutic target for diseases in both the central nervous system and periphery. The current study aimed to develop and evaluate a suitable carbon-11 labeled tracer for imaging MAGL in preclinical studies.

**Methods:**

(*R*)-YH168 was synthesized via a multi-step pathway and its half-maximal inhibitory concentration (*IC*_50_) values were measured using an enzymatic assay. Radiosynthesis of (*R*)-[^11^C]YH168 was accomplished by ^11^C-methylation via Suzuki cross-coupling of a pinacol boron precursor. In vitro autoradiography was performed using brain tissues from MAGL knockout and the corresponding wild-type mice. The metabolic stability of (*R*)-[^11^C]YH168 in mouse brain and plasma was assessed 5 min after injection. Dynamic PET scans were conducted on anesthetized mice and rhesus monkey. For studies in non-human primates, arterial blood samples were analyzed to obtain the input function for kinetic modeling. Blocking studies with the irreversible MAGL inhibitor PF-06795071 were performed to assess the binding specificity of (*R*)-[^11^C]YH168.

**Results:**

(*R*)-[^11^C]YH168 was synthesized *via* Suzuki coupling of the phenyl boronic ester with [^11^C]CH_3_I in the presence of palladium catalyst. In vitro autoradiography revealed a heterogeneous distribution pattern of (*R*)-[^11^C]YH168 with higher binding to MAGL-rich brain regions in wild-type mouse brain slices compared to that of MAGL knockout mice. Dynamic PET imaging in wild-type and MAGL knockout mice confirmed its high specificity and selectivity in mouse brains. In the rhesus monkey, (*R*)-[^11^C]YH168 displayed good brain permeability. High levels of radioactivity uptake were seen in the cingulate cortex, frontal cortex, cerebellum, occipital cortex, and hippocampus, consistent with MAGL expression. The one-tissue compartment model was appropriate for fitting the regional time-activity curves and provided reliable volume of distribution values across all brain regions. Pretreatment with PF-06795071 (0.1 mg/kg) resulted in almost complete blockade (> 95%) of radioactivity uptake, demonstrating binding specificity of (*R*)-[^11^C]YH168 to MAGL in the non-human primate brain. The regional non-displaceable binding potential follows the rank order of cingulate cortex ~ frontal cortex ~ insula > putamen > temporal cortex > caudate ~ occipital cortex ~ thalamus > nucleus accumbens ~ hippocampus ~ cerebellum ~ globus pallidus > substantia nigra > amygdala.

**Conclusion:**

(*R*)-[^11^C]YH168 is a promising PET probe for imaging and quantifying MAGL in the brains of mice and non-human primates. This ^11^C-labeled tracer holds great potential for translation into human subjects and offers the possibility of performing multiple PET scans on the same subject within a single day.

**Supplementary Information:**

The online version contains supplementary material available at 10.1007/s00259-024-07013-0.

## Introduction

Human monoacylglycerol lipase (MAGL) consists of 303 amino acids and belongs to the serine hydrolase enzyme family [1]. This enzyme is located at the presynaptic terminal of neurons and plays a key role in the degradation of 2-arachidonoylglycerol (2-AG), an endocannabinoid that traverses the synaptic cleft to activate cannabinoid receptor 1 [2]. Pharmacological inhibition of MAGL leads to elevated levels of 2-AG in the central nervous system (CNS) and mitigates inflammatory biomarkers such as prostaglandin E2, interleukin-1β, and tumor necrosis factor-α [3]. Consequently, the development of selective and potent MAGL inhibitors has gained considerable attention for treating neurological disorders [4]. To date, ABX-1431, developed by Lundbeck (Abide therapeutics), has been evaluated in various phase I clinical trials for the treatment of hyperalgesia, functional dyspepsia, Tourette syndrome, central pain and neuropathic pain [5].

As a non-invasive imaging technique, positron emission tomography (PET) has become an irreplaceable tool to quantify drug-target interactions during drug development. Fluorine-18 with a half-time (t_1/2_) of 109.8 min, and carbon-11, with a t_1/2_ of 20 min, are the most commonly used isotopes in molecular probes for PET neuroimaging. Previously, we developed (*R*)-[^18^F]YH134 for mapping MAGL in rodent brains and successfully demonstrated its potential applications in both central nervous system (CNS) and peripheral drug development [6]. Although the short t_1/2_ of carbon-11 may limit its clinical application, it also allows for adaptive study design in a biomedical imaging center with an on-site cyclotron. This is particularly beneficial for studying therapeutic drug candidates that interact with multiple targets, such as Centanafadine, an inhibitor of norepinephrine (NET), dopamine (DAT), and serotonin (SERT) reuptake transporters. Its phase I drug occupancy studies in healthy male adults were completed in a relatively short time frame and with a small cohort due to the availability of two target-specific ^11^C-labeled tracers ([^11^C]MRB for NET; [^11^C]DASB for SERT) and an ^18^F-labeled tracer [^18^F]FE-PE2I for DAT [7]. Thus, the aim of the current study was to develop a reversible ^11^C-labeled MAGL PET tracer capable of quantifying MAGL expression in non-human primate (NHP), with the potential to ultimately be translated into human studies.

**Fig. 1:**
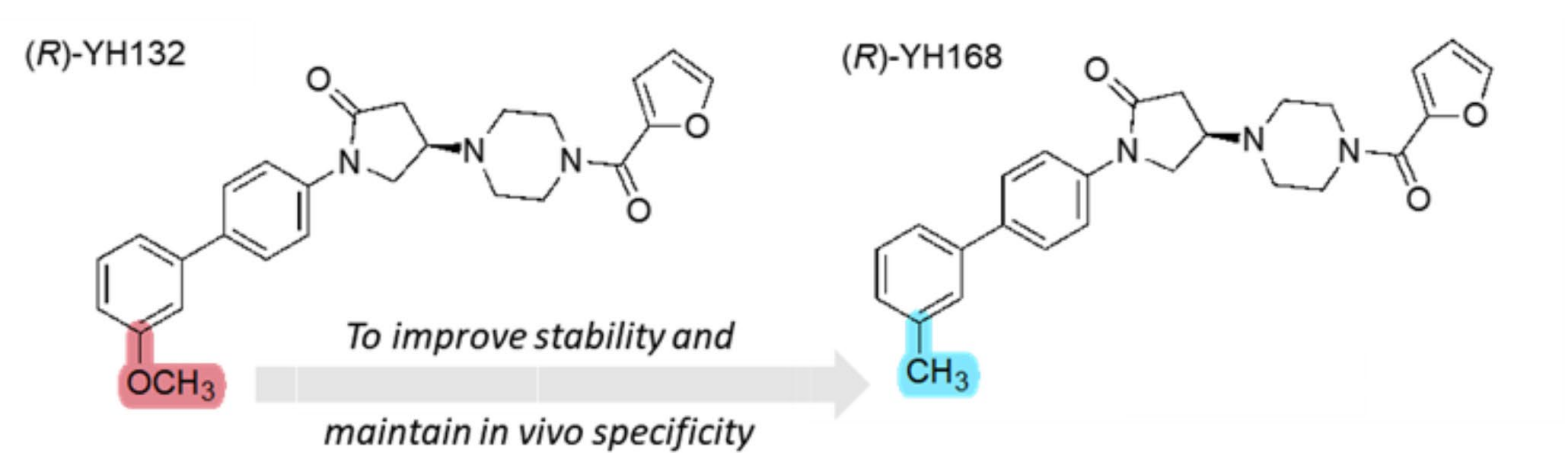
Chemical structures of (*R*)-YH132 and (*R*)-YH168

In our previous attempt to develop a suitable ^11^C-labeled PET tracer for MAGL occupancy studies, (*R*)-[^11^C]YH132 (Fig. 1) was synthesized via *O*-methylation using [^11^C]CH_3_I and was evaluated in pre-clinical studies [8]. Despite its in vivo specificity, its practical application is hindered by the observation that only 85% of the tracer is intact in mouse brain homogenates 5 min post-injection (Supplemental Fig. S1). To address this limitation, we developed (*R*)-YH168, a toluene derivative of (*R*)-YH132. We anticipate that (*R*)-YH168 will provide increased in vivo stability and at the same time maintain high MAGL binding affinity (Fig. 1). Its radiolabeling could be accomplished by coupling of the corresponding phenylic boronic esters with [^11^C]CH_3_I in the presence of a palladium complex [9]. The specificity and selectivity of (*R*)-[^11^C]YH168 were evaluated by in vitro autoradiography using brain sections from MAGL knockout and wild-type mice. Dynamic PET imaging and metabolite studies were conducted in mice. Subsequently, PET studies with (*R*)-[^11^C]YH168 were carried out in a rhesus monkey to quantitatively assess its pharmacokinetic and imaging characteristics.

## Materials and methods

### Chemistry

The synthesis of phenylboronic ester precursor was carried out as previously described [10]. Enantiomerically pure (*R*)-YH168 was synthesized in analogy to (*R*)-YH132, and the corresponding synthetic route is presented in the supporting information (Supplemental Scheme 1). Chiral separation by supercritical fluid chromatography (SFC) (ChiralPak IH, 5 μm, 250 × 20 mm, 45% MeOH in CO_2_) was performed on the final racemic product to obtain (*R*)-YH168 in 98.8% ee. ^1^H NMR (400 MHz, CDCl_3_) δ 7.75–7.54 (m, 4 H), 7.49 (s, 1H), 7.42–7.29 (m, 3 H), 7.16 (d, J = 7.3 Hz, 1H), 7.05 (s, 1H), 6.50 (s, 1H), 4.26–3.66 (m, 6 H), 3.38 (s, 1H), 3.01–2.47 (m, 6 H), 2.42 (s, 3 H). HRMS (ESI) calculated for C_26_H_28_N_3_O_3_^+^ [M + H]^+^, 430.2125 m/z; found, 430.2120 m/z.

### Characterization of (*R*)-YH168

The half-maximal inhibitory concentration (*IC*_50_) of (*R*)-YH168 was measured using human, cynomolgus monkey or mouse MAGL protein according to the published procedure [11]. Microsomal clearance using commercially available pooled liver microsomes (C57BL/6J mice) and parallel artificial membrane permeability assay (PAMPA) were conducted as previously reported [12].

### Radiochemistry

[^11^C]CH_3_I was bubbled into the reaction vial with 0.5 mg precursor, 0.5 mg [Pd_2_(dba)_3_], 0.5 mg P(*o*-tolyl)_3_, and 0.6 mg K_2_CO_3_ in the mixture of DMF/H_2_O (v/v = 35/4, 390 µL). The reaction was heated at 65 °C for 4 min. After dilution with 0.1% H_3_PO_4_ in H_2_O/MeCN (v/v = 1/1, 1.7 mL), the resulting mixture was loaded to a semi-preparative HPLC for purification (Phenomenex Gemini C18 column, 250 mm × 10 mm, 5 μm, 110 Å, mobile phase A: 0.1% H_3_PO_4_ in H_2_O, mobile phase B: MeCN, gradient with 0–6 min, 10–35% B; 6–8 min, 35–45% B; 8–12 min, 45% B; 12–18 min, 45–50% B; 18–20 min, 50–95% B with a flow of 4 mL/min). The fraction corresponding to the product was collected, diluted with 8 mL of water, and passed through a pre-conditioned C18 light SepPak cartridge (Waters, WAT023501), followed by rinsing with 5 mL of water. The product was eluted off the cartridge with 0.5 mL of EtOH, and formulated with phosphate-buffered saline (9.5 mL, Gibco) to give a neutralized solution (pH = 7.4). The identity of the tracer was confirmed by co-injection with the reference compound using the Agilent 1100 series HPLC system equipped with a UV detector, and a GabiStar radiodetector (Raytest) (ACE XDB-C18 Zobrax column, 75 mm×4.6 mm, 3.5 μm, mobile phase A: 0.1% H_3_PO_4_ in H_2_O, mobile phase B: MeCN, gradient with 0.0–3.0 min, 5 − 30% B; 3.0–4.0 min, 30 − 40% B; 4.0–6.0 min, 40 − 50% B; 6.0–9.0 min, 50 − 95% B with a flow of 1 mL/min, UV detection at 254 nm). The enantiomeric purity of the final product was evaluated by a chiral column (ChiralCel OD, 5 μm, 250 mm×4.6 mm, mobile phase: hepatane/0.5%TEA IsoPrOH = 1/1, v/v with a flow of 2 mL/min). Representative HPLC chromatograms using the chiral column are presented in Supplementary Fig. 2.

### In vitro evaluation

For the assessment of plasma stability, (*R*)-[^11^C]YH168 (10 µL, 1‒2 MBq) was added to mouse plasma (300 µL). The mixture was incubated at 37 °C with gentle shaking. At 5, 30 and 60 min, 100 µL of the sample were taken out and mixed with ice-cold acetonitrile (200 µL). After centrifugation, the supernatants were filtered and analysed by HPLC equipped with column-switching system. Briefly, the sample was transferred to a pre-column (ReproSil-Pur, 120 ODS-3, 10 μm, 20 × 4.6 mm, Dr. Maisch GmbH, Germany) using 1% MeCN in H_2_O (1 mL/min, 0–4 min), and subsequently eluted and analyzed using Luna C18 column (5 μm, 250 × 4.6 mm, Phenomenex Inc., Germany) with 55% MeCN in H_2_O (1 mL/min, 4–15 min) as mobile phase. The UV and radioactive signals were detected by 220 nm diode array detector L-2450 (Hitachi High-Technologies, Japan) and FlowStar, LB 513 (Berthold Technologies GmbH & Co. KG, Germany), respectively. The data were corrected for physical decay, integrated and analyzed using the EZ Chrome Elite Software Package (Version 3.3.1, Agilent Technologies Inc., United States). In vitro autoradiography and free fraction measurement in mouse plasma were conducted as previously reported [10].

### In vivo evaluation in mice

MAGL knockout (*n* = 3) and wild type (*n* = 3) mice were administrated with 8.02‒14.6 MBq (*R*)-[^11^C]YH168 (5.78‒10.71 nmol/kg) *via* tail vein injection for PET imaging. Data acquisition and reconstruction were performed as previously described [12]. The time-activity curves were generated with predefined volumes of interest using an MRI T2 template by PMOD software (version 4.201; PMOD Technologies, Fällanden, Switzerland).

For in vivo metabolite analysis, (*R*)-[^11^C]YH168 was injected intravenously to wild-type mice, and the animals were sacrificed at 5 and 30 min post-injection. After decapitation, blood was rapidly collected in a tube (BD Vacutainer, LH Lithium heparin) and mixed. The blood samples were centrifuged at 5000 RCF for 3 min at 4 °C to separate the plasma. The supernatant was collected, mixed with equal volume of cold acetonitrile and centrifuged at 5000 RCF for 3 min at 4 °C for deproteinization. The brain was dissected and homogenized using a polytron (PT 2100) in PBS and cold acetonitrile was added (v/v = 1/1 in the final mixture). The homogenate was vortexed and centrifuged at 4800 rpm for 5 min at 4 °C. All supernatants were aspirated with a syringe and filtered through a 0.45 μm filter unit (Whatman, SPARTAN 13/0.45 RC). The resulting samples were analyzed by the column-switching HPLC system as mentioned above. The in vitro stability of (*R*)-[^11^C]YH168 in brain homogenates was further examined by co-incubating the tracer with freshly prepared brain tissue at 37 °C. Samples were collected at 5 and 30 min after incubation and analyzed as previously described.

### PET studies in rhesus monkey

Two scans were performed in one monkey, a baseline scan to evaluate the kinetic properties, and a blocking scan with MAGL inhibitor PF-06795071 at 0.1 mg/kg dose to evaluate the binding specificity. The radiotracer or blocking drug was given as a 3 min bolus injection by an infusion pump, and a dynamic PET scan was conducted on the Focus 220 scanner (Siemens Medical Solutions, Knoxville TN USA) as previously reported [13]. The data acquisition lasted for 120 min. Arterial blood samples were collected at preselected time points, separated and measured in a gamma counter (Wizard 1480/2480, PerkinElmer, Waltham, MA, USA) to obtain the radioactive uptake in whole blood and plasma overtime. Heart rate, blood pressure, respirations, SpO_2_, electrocardiogram, end-tidal CO_2_, and body temperature of the animal were continuously monitored during the scan.

For metabolite analysis, arterial blood samples at 0, 3-, 8-, 15-, 30-, 60- and 90-min post-injection were centrifuged at 3900 g at 4 °C for 5 min to separate the plasma. The supernatant was collected, mixed with 8 M urea to denature plasma proteins and passed through a 1.0 μm filter (Whatman 13 mm CD/X). The filtrate was then analyzed by HPLC with a column-switching system. Self-packed column (4.6 × 19 mm with C18 sorbent) and Phenomenex Luna C18 (2) (4.6 × 250 mm, 5 μm) were used for capture and analysis, respectively. The mobile phase consisted of 55% MeCN in 45% 0.1 M ammonium formate (pH = 6.4, v/v) at a flow rate of 1.4 mL/min. The eluent fractions were collected with an automated fraction collector. The radioactivity in the whole blood, plasma, filtered plasma-urea mix, filter and collected fractions were measured in a gamma counter. The arterial plasma input function (AIF) was calculated and corrected by the percentages of parent tracer, filtration efficiency and the ratio of plasma/whole blood.

### Image processing and kinetic modeling

Data reconstruction was carried out using a Fourier rebinning and filtered back projection algorithm. High-resolution magnetic resonance image (Siemens 3T Trio) was registered to the PET images and used as a template to draw regions of interest (ROIs). The time activity curves (TACs) of ROIs overtime were generated. Regional TACs were fitted into one-tissue and two-tissue compartment (1TC, 2TC) models [14]. Akaike information criterion (AIC) was used to evaluate the goodness-of-fits of three models [15].

Regional distribution volume (*V*_T_, mL/cm^3^) was calculated from kinetic analysis. The occupancy plot was used to obtain MAGL occupancy by PF-06795071 (0.1 mg/kg) and non-displaceable volume of distribution (*V*_ND_) of (*R*)-[^11^C]YH168 [16]. Non-displaceable binding potential (*BP*_ND_) was calculated from regional *V*_T_ under control conditions and *V*_ND_, i.e. *BP*_ND_ = (*V*_T_, _ROI_ - *V*_ND_)/ *V*_ND_ [17].

## Results

### Characterization of (*R*)-YH168

Racemic YH168 was synthesized *via* Suzuki coupling and subsequent chiral separation by SFC afforded enantiomerically pure (*R*)-YH168. The enantiomeric excess value of (*R*)-YH168 was determined to be 98.8% by SFC-mass spectrometry using a chiral column. IC_50_ values of (*R*)-YH168 toward mouse, cynomolgus monkey and human MAGL were 3.8, 2.7 and 4.0 nM, respectively.

### Radiochemistry

(*R*)-[^11^C]YH168 was obtained *via* a single-step ^11^C-methylation of the phenyl boronic ester with [^11^C]CH_3_I in the presence of K_2_CO_3_ and [Pd_2_(dba)_3_]/P(*o*-tolyl)_3_, as shown in Scheme 2. The total synthesis time was around 40 min. (*R*)-[^11^C]YH168 was obtained with a radiochemical yield of 26 ± 6% (decay-corrected, *n* = 6). The radiochemical purity of the final product was greater than 99%. At the end of radiosynthesis, the molar activity of the tracer was measured in the range of 64–99 GBq/µmol (*n* = 6). The identity of (*R*)-[^11^C]YH168 was confirmed by co-injection with the non-radioactive (*R*)-YH168 into analytical radio-high-performance liquid chromatography (radio-HPLC).

**Fig. 2:**
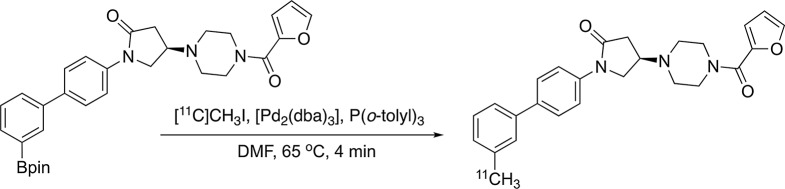
Radiosynthesis of (*R*)-[^11^C]YH168

### In vitro and in vivo evaluation in mice

**Fig. 3:**
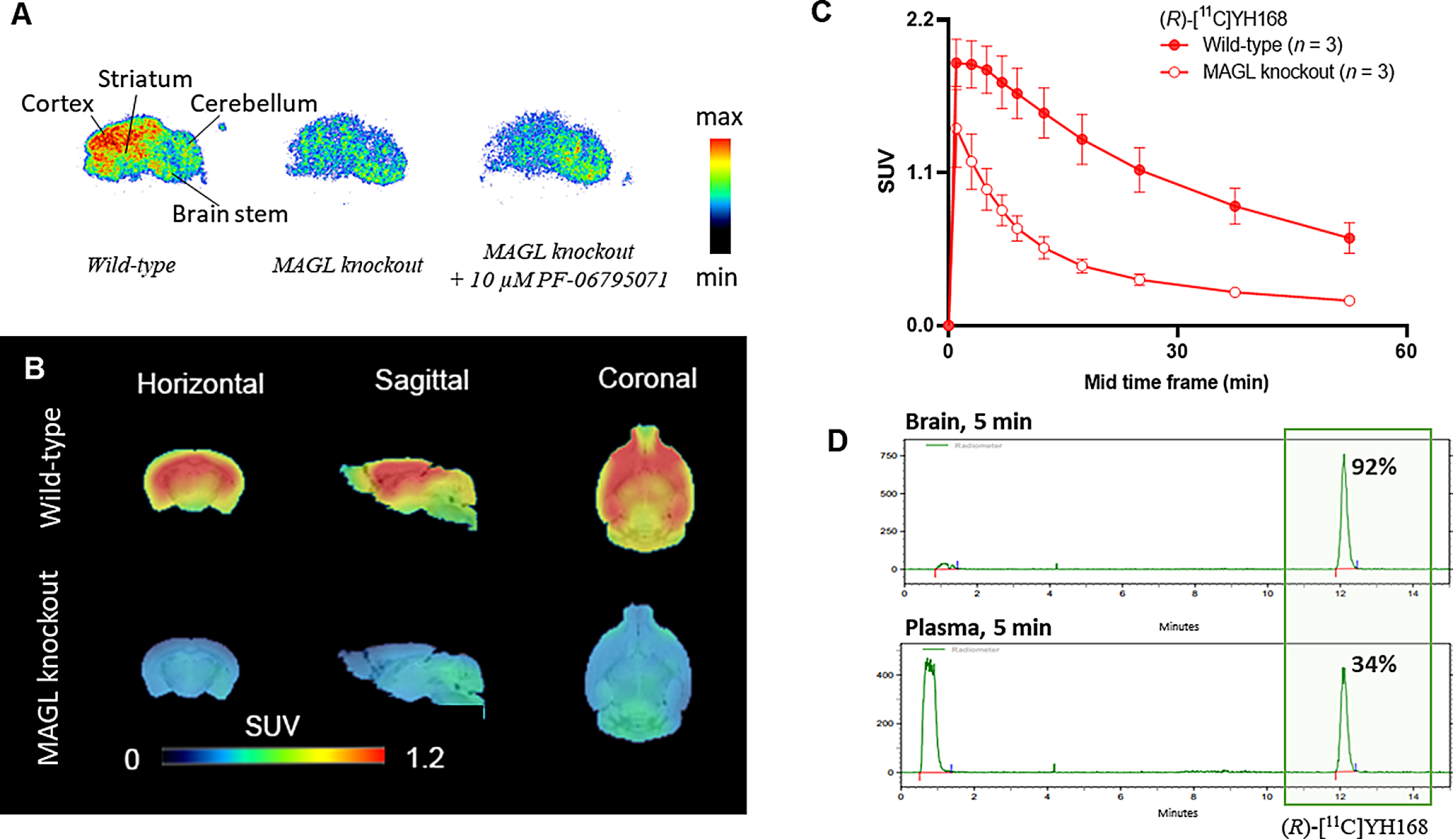
(**A**) In vitro autoradiograms and (**B**) in vivo brain PET images of (*R*)-[^11^C]YH168 averaged from 9.0 to 60 min post injection in wild-type and MAGL knockout mice as indicated. SUV, standardized uptake value. PET data overlaid on an MRI template. (**C**) Time activity curves (TACs) of (*R*)-[^11^C]YH168 in the whole brain. (**D**) Ex vivo radiometabolite analysis of (*R*)-[^11^C]YH168 in the brain homogenate and plasma of wild-type mouse at 5 min post-injection

Incubation of (*R*)-[^11^C]YH168 (~ 2 nM) with wild-type mouse brain sections revealed a heterogeneous distribution of radioactive accumulation. As shown in Fig. 3A, high levels of radioactivity were found in the cortex and striatum, whereas moderate signals were detected in the cerebellum and brain stem, which aligns with the MAGL expression pattern in rodents [18]. By contrast, brain tissues of MAGL knockout mice revealed significantly reduced radioactivity in MAGL-rich brain regions, resulting in a homogenous radioactivity distribution in the whole brain. Co-incubation of the radiotracer with 10 µM PF-06795071, a high-affinity irreversible MAGL inhibitor, did not lead to further reduction of radioactive signals in brain tissues of MAGL knockout mice. These findings confirm the in vitro specificity and selectivity of the novel tracer. When incubated with mouse plasma for 60 min, the radiochemical purity of the product remained above 99%, indicating its in vitro stability in blood. The free fraction of (*R*)-[^11^C]YH168 was determined to be 14.7 ± 0.2% (*n* = 3) in mouse plasma.

Representative PET images of (*R*)-[^11^C]YH168 in MAGL knockout and wild-type mouse brains averaged from 9.0 to 60 min post-injection are displayed in Fig. 3B. In agreement with its autoradiograms in vitro, high radioactivity uptake was evident in the cortex, striatum and hippocampus of wild-type mouse, while much lower radioactivity level was seen in MAGL knockout mouse brains. Figure 3C depicts the corresponding time activity curves (TACs) of (*R*)-[^11^C]YH168 in mouse brains. In wild-type mouse brains, the maximal standardized uptake value (SUV = 1.89 ± 0.17, *n* = 4) peaked at 1 min post-injection, followed by a gradual washout over time. In contrast, the radiotracer exhibited rapid washout with notably shorter retention time in MAGL knockout mouse brains, indicating high specificity of the novel tracer. As shown in Figs. 3D and 92% of the radioactive signal in brain homogenates was attributed to (*R*)-[^11^C]YH168 at 5 min post-injection. In plasma sample, the tracer was rapidly degraded and a predominant radiometabolite with a retention time of less than 2 min was detected.

### PET studies in rhesus monkey

The injected dose of (*R*)-[^11^C]YH168 was 174 and 147 MBq, respectively (cold mass of 0.057 and 0.047 µg/kg) for the baseline and blocking studies, with molar activity of 116 and 119 MBq/nmol at the end of synthesis.

#### Plasma analysis

**Fig. 4:**
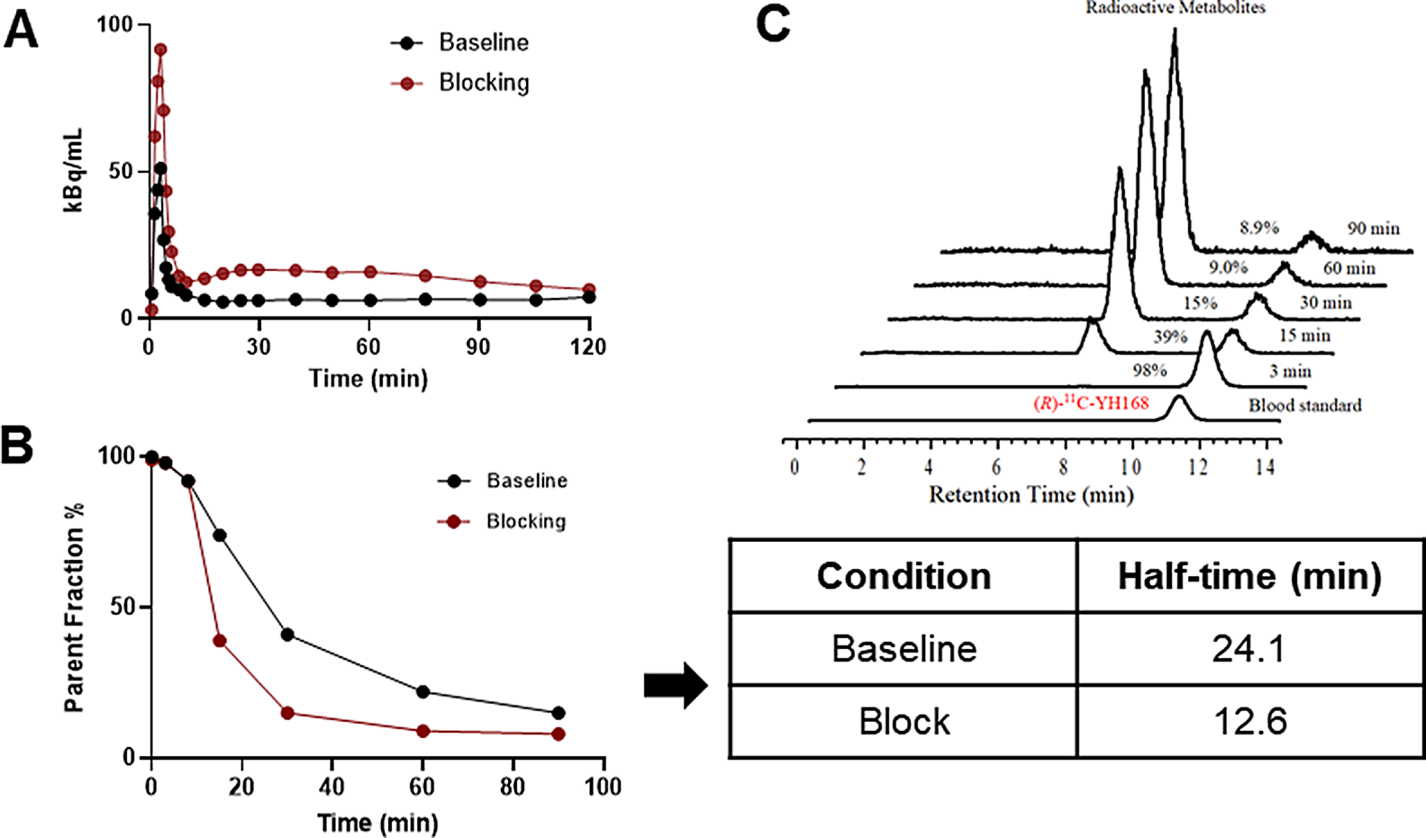
(**A**) Decay-corrected plasma activity overtime. (**B**) Parent fraction of (*R*)-[^11^C]YH168 at different time points under baseline and blockade conditions. (**C**) Radioactive metabolite profiles of (*R*)-[^11^C]YH168, arterial blood samples were analyzed by column-switching HPLC system

After the bolus injection of (*R*)-[^11^C]YH168, the radioactivity in plasma reached maximal uptake within 3 min (Fig. 4A), followed by fast clearance. Notably, pretreatment with a 0.1 mg/kg dose of PF-06795071, an irreversible MAGL inhibitor, resulted in increased radioactivity in plasma. The percentages of the parent tracer decreased over time, as shown in Fig. 4B. The data were fitted by nonlinear fit in Prism (one-phase decay, version 9.4.1). The half-life of the intact tracer under baseline condition was determined to be 24.1 min, while it decreased to 12.6 min with PF06795071 as a competitor. Representative chromatograms from column-switching HPLC are depicted in Fig. 4C. At 15 min post-injection, the percentage of (*R*)-[^11^C]YH168 declined to 39%, coinciding with the emergence of a polar radioactive metabolite. This metabolite with a retention time of ~ 6.76 min gradually increased overtime and became the predominant radioactive species from 30 min post-injection.

#### Brain imaging

**Fig. 5:**
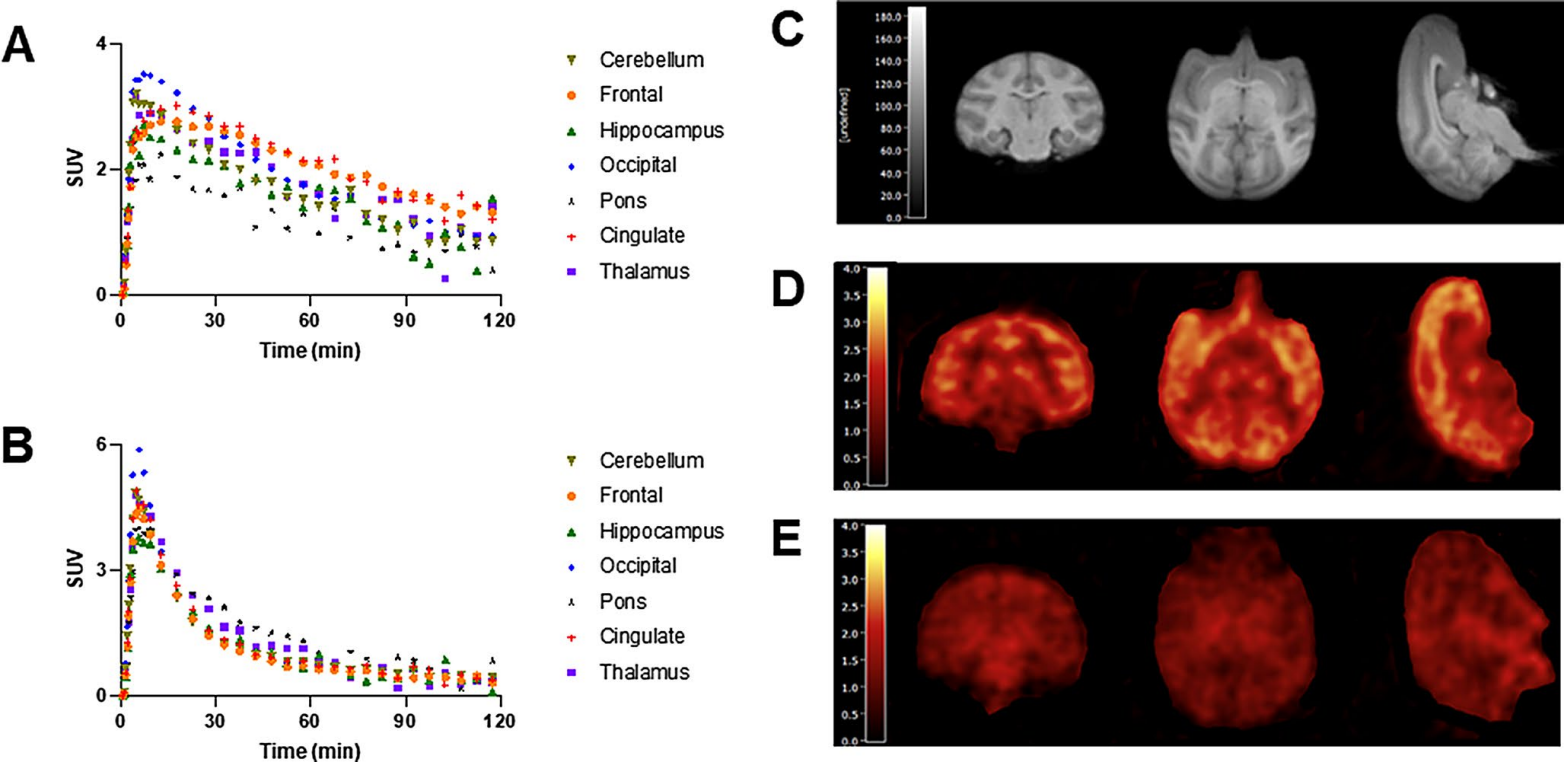
Time-activity curves in selected brain regions of NHP from the baseline (**A**) and blocking (**B**) scans. (**C**) Representative MR Image for ROI segmentation. (**D**) PET images averaged from 30 to 45 min after (*R*)-[^11^C]YH168 injection under baseline (tracer only), and (**E**) blockade conditions (tracer and 0.1 mg/kg PF-06795071)

The corresponding regional brain TACs in the baseline and blocking scans are shown in Fig. 5A and B. Figure 5C illustrates the MR image used for defining regions of interest (ROIs), and PET images (Fig. 5D and E) averaged from 30 to 45 min after the injection of (*R*)-[^11^C]YH168, obtained from the baseline and blocking scans. In the baseline scan, the tracer entered the brain quickly and reached maximal standardized uptake value (SUV_max_) within 10 min post-injection (Fig. 5A). The SUV_max_ was between 2.0 and 2.5 for cerebellum, frontal cortex, cingulate cortex and occipital cortex, whereas a relatively lower peak radioactivity was found for pons (SUV_max_ ~1.5). As shown in Fig. 5D, (*R*)-[^11^C]YH168 exhibited a heterogeneous distribution in the NHP brain with high uptake in MAGL-rich brain regions and low uptake in pons and white matter. Pretreatment with PF-06795071 (0.1 mg/kg, iv) 10 min prior to the administration of the radiotracer induced a rapid washout of (*R*)-[^11^C]YH168 from the brain (Fig. 5B) and resulted in homogeneous PET brain images with a significantly reduced accumulation of radioactivity (Fig. 5E).These results suggest specific and selective binding of tracer in the monkey brain.

#### Kinetic modeling

**Fig. 6:**
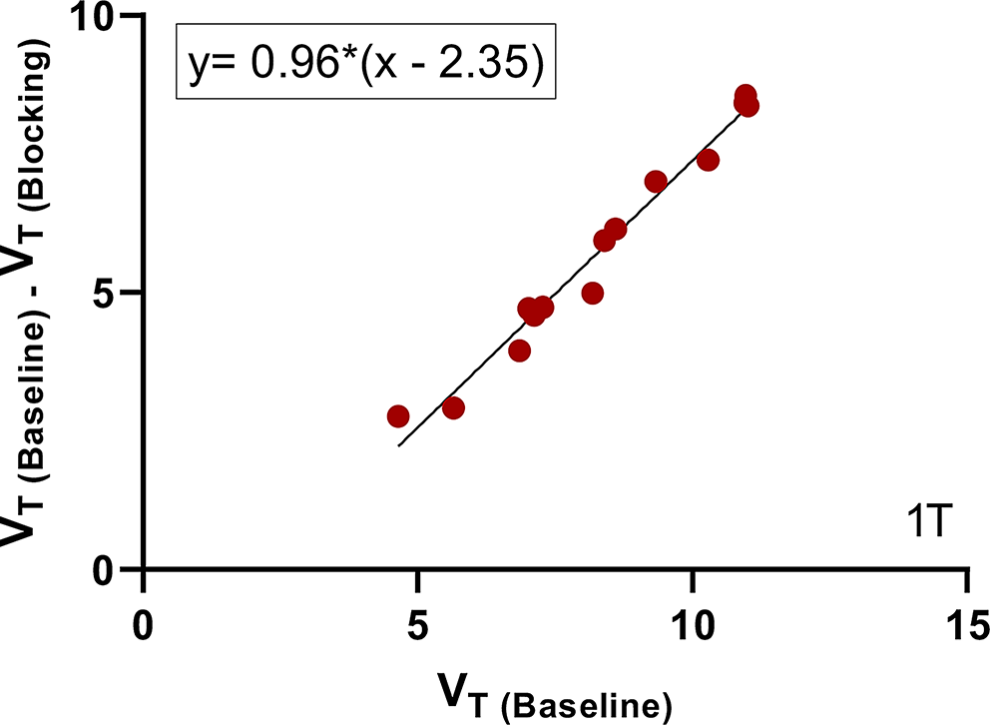
Lassen plot of (*R*)-[^11^C]YH168 with and without pretreatment of PF-06795071 (0.1 mg/kg, iv) using results from the 1TC model. The slope of the linear regression is the estimated MAGL occupancy by in vivo competitor, and the x-intercept/slope is used for the estimation of non-displaceable volume of distribution (*V*_ND_)

Using the metabolite-corrected arterial input function, regional TACs were processed with both the 1TC and 2TC models to generate binding parameters. The 1TC model provided better fits than the 2TC model, yielding reliable *V*_T_ estimates across various brain regions. Regional distribution volumes (*V*_T_) estimated from the 1TC model are listed in Table 1. Under baseline conditions, high *V*_T_ values were observed in the brain regions with high levels of MAGL, such as the cingulate cortex, frontal cortex, temporal cortex, putamen, hippocampus, and insula, followed by moderate *V*_T_ values in the caudate, cerebellum and thalamus. Pretreatment with PF-06795071 (0.1 mg/kg) significantly reduced *V*_T_ values of (*R*)-[^11^C]YH168 in all brain regions, suggesting the lack of suitable reference region for MAGL in NHP. Based on Lassen plot analysis, the specific binding sites of (*R*)-[^11^C]YH168 were saturated by the potent MAGL inhibitor PF-06795071 at a dose of 0.1 mg/kg (Fig. 6). The non-displaceable volume of distribution (*V*_ND_) of 2.35 mL/cm^3^ was derived from the Lassen plot, calculated as the *x*-intercept divided by the slope in Fig. 6. The regional non-displaceable binding potential (*BP*_ND_) values were then calculated using the formula: *BP*_ND_ = (*V*_T_ - *V*_ND_)/*V*_ND_ and are summarized in Table 1. The *BP*_ND_ values of the novel tracer across different brain regions followed the rank order: cingulate cortex ~ frontal cortex ~ insula > putamen > temporal cortex > caudate ~ occipital cortex ~ thalamus > nucleus accumbens ~ hippocampus ~ cerebellum ~ globus pallidus > substantia nigra > amygdala.

**Table 1:** 1TC-derived regional volume of distribution (*V*_T_) and nondisplaceable binding potential (*BP*_ND_) values of (*R*)-[^11^C]YH168 in the monkey brain

	1TC-derived V_T_ (mL/cm^3^)		BP_ND_
Brain region	Baseline	Blockade	
Amygdala	4.64	1.89	0.98
Caudate	8.61	2.46	2.66
Cerebellum	7.02	2.31	1.99
Cingulate	11.01	2.64	3.69
Frontal cortex	10.97	2.42	3.67
Globus pallidus	6.85	2.90	1.92
Hippocampus	7.12	2.53	2.03
Insula	10.95	2.53	3.66
Nucleus accumbens	7.28	2.55	2.10
Occipital cortex	8.40	2.46	2.57
Putamen	10.29	2.90	3.38
Substantia nigra	5.65	2.74	1.40
Temporal cortex	9.34	2.33	2.97
Thalamus	8.18	3.20	2.48

## Discussion

The extracts from cannabis (C. sativa or indica) have been recorded for the treatment of human diseases for centuries. The approval of nabiximols, a combination of tetrahydrocannabinol and cannabidiol, for alleviating symptoms in multiple sclerosis has brought the limelight to the endocannabinoid system in treating neurological disorders [19]. However, the complex interaction of endocannabinoid components and their ubiquitous expression throughout the body has posed challenges in therapeutic drug development [20, 21]. This is exemplified by the severe neurological disorder that occurred during a phase I trial of the fatty acid amide hydrolase (FAAH) inhibitor BIA 10-2474 [22] and the psychiatric side effects associated with the cannabinoid receptor 1 (CB1) inverse agonist rimonabant [23]. Of note, molecular probes labeled with carbon-11 offer advantages for efficiently conducting drug occupancy studies on various targets. Together with the well-developed ^11^C-labeled PET tracers targeting FAAH ([^11^C]MK3168) [24] and CB1 ([^11^C]OMAR) [25], we believe that the development of ^11^C-labeled MAGL tracer could be potentially valuable to accelerate drug discovery in the endocannabinoid system.

Characterizations of (*R*)-YH132 and (*R*)-YH168 are summarized in Supplementary Table 1. In in vitro inhibition assay, YH168 displayed low nanomolar affinity towards mouse, NHP and human MAGL. This is consistent with previous findings that structural modification in the lipophilic binding pocket is well-tolerated [10]. Comparing whole brain TACs of (*R*)-[^11^C]YH132 and (*R*)-[^11^C]YH168 in MAGL knockout and wild-type mice (Supplementary Fig. 3), we found that switching from methoxy to methyl substitution resulted in increased brain uptake in wild-type mice (1.89 ± 0.17 SUV vs. 1.33 ± 0.18 SUV at 1 min post-injection). This difference may be related to the slightly higher lipophilicity and increased brain permeability of (*R*)-[^11^C]YH168. Meanwhile, a rapid and steady washout from MAGL knockout brains was demonstrated by (*R*)-[^11^C]YH168, resulting in lower non-specific radioactivity accumulation in mouse brains compared to (*R*)-[^11^C]YH132. Moreover, (*R*)-[^11^C]YH168 with C_sp2_–[^11^C]CH_3_ displayed superior in vivo stability in brain homogenates than (*R*)-[^11^C]YH132 with C_sp2_–*O*–[^11^C]CH_3_ (Supplementary Table 1). We additionally conducted ex vivo radiometabolite studies 30 min after the administration of (*R*)-[^11^C]YH168. In addition to 40% intact compound in the brain sample, a polar radiometabolite with a retention time of 0.65 min was observed (Supplementary Fig. 4). Since the polar radiometabolite is less likely to cross the blood-brain barrier and it is the major radioactive species in the blood sample, (*R*)-[^11^C]YH168 was further incubated with freshly prepared C57/Bl6 mouse brain homogenates at 37 °C to investigate whether this polar radiometabolite is generated in the brain. As shown in Supplementary Fig. 5, (*R*)-[^11^C]YH168 is the only detectable radioactive species in the analysis. We therefore concluded that the polar radiometabolite identified in the brain homogenates may be contaminated by the blood.

Encouraged by the preliminary results obtained in mice, PET studies of (*R*)-[^11^C]YH168 were further carried out in NHP with arterial input function. In rhesus monkey, (*R*)-[^11^C]YH168 was metabolized at a moderate rate, whereas blocking with PF-06795071 accelerated the process. A much polar radioactive metabolite was detected, which is unlikely to cross the blood-brain barrier and therefore does not require consideration in quantitative PET analysis. Peak uptake was reached within 10 min under baseline and blocking conditions in all brain regions. It is noteworthy that pretreatment with PF-06795071 at a dose of 0.1 mg/kg induced a higher initial brain penetration. This may be attributed to the blockade of MAGL in the periphery, leading to an increased concentration of free tracer in the bloodstream, as suggested in our previous work [6].

In the quantitative kinetic analysis of the NHP data, the 1TC model provided good fits to the regional TACs and was chosen as the appropriate model to calculate the underlying binding parameters. Regional *V*_T_ values showed high binding of the radiotracer in the frontal cortex, cingulate cortex, temporal cortex and putamen, and low binding in the pons, consistent with PET imaging results of MAGL PET tracer [^18^F]T-401 in NHPs [26]. Under blocking conditions, reduced *V*_T_ values were observed in all regions, suggesting the absence of a reference region. Further clarification of the specific binding signals of the tracer was obtained through *BP*_ND_ values. (*R*)-[^11^C]YH168 yielded *BP*_ND_ values > 0.5 in most brain regions, indicating its feasibility for accurate estimation in quantitative PET kinetic modeling [27, 28].

## Conclusion

We have successfully developed a reversible ^11^C-labeled MAGL PET tracer, (*R*)-[^11^C]YH168, and conducted a detailed evaluation in mice and a nonhuman primate. Compared to (*R*)-[^11^C]YH132, it demonstrated improved kinetic and binding profiles in mice, characterized by increased brain uptake, high in vivo specificity and stability. With promising properties observed in non-human primates, including appropriate kinetics and reliable *BP*_ND_ estimates in different brain regions, we believe that (*R*)-[^11^C]YH168 holds great potential to serve as a suitable and effective neuroimaging PET tracer for MAGL.

## Electronic supplementary material

Below is the link to the electronic supplementary material.

Supplementary Material 1

## Data Availability

The datasets generated and analyzed in this study are available on request from the corresponding author.
