# Supplementary material for: Exploration of (R)-[11C]YH168 as a PET tracer for imaging monoacylglycerol lipase in the brain: from mice to non-human primates
Source: Eur J Nucl Med Mol Imaging. 2024 Dec 14;52(4):1556–65. doi: 10.1007/s00259-024-07013-0 (PMC11839854; doi:10.1007/s00259-024-07013-0)
Supplement: Supplementary file 1 — Supplementary Material 1 [file 259_2024_7013_MOESM1_ESM.docx]

**Supplemental Data for**

**Exploration of (*R*)-[^11^C]YH168 as a PET tracer for imaging monoacylglycerol lipase in the brain: from mice to non-human primates**

Yingfang He^1, 4#^, MingQiang Zheng^2#^, Jiwei Gu^2^, Lisa Reichert^1^, Johannes Trimborn^1^, Hui Zhang^2^, Claudia Keller^1^, Mallory Crosby^2^, Ludovic Collin^3^, Dominik Heer^3^, Anto Pavlovic^3^, Andreas Topp^3^, Matthias Wittwer^3^, Uwe Grether^3^, Luca Gobbi^3^, Roger Schibli^1^, Henry Huang^2*^, Linjing Mu^1*^

^1^ Center for Radiopharmaceutical Sciences, Institute of Pharmaceutical Sciences, Department of Chemistry and Applied Biosciences, ETH Zurich, CH-8093 Zurich, Switzerland

^2^ Yale PET Center, Department of Radiology and Biomedical Imaging, Yale University, New Haven, Connecticut

^3^ Pharma Research and Early Development, Roche Innovation Center Basel, F. Hoffmann-La Roche Ltd, CH-4070 Basel, Switzerland

^4^ Institute of Radiation Medicine, Fudan University, Xietu Road 2094, Shanghai 200032, China

^#^Yingfang He and MingQiang Zheng contributed equally to this work

**^*^Corresponding author**

Henry Huang

E-mail address: [henry.huang@yale.edu](mailto:henry.huang@yale.edu)

Linjing Mu (https://orcid.org/0000-0001-5354-1546)

E-mail address: [linjing.mu@pharma.ethz.ch](mailto:linjing.mu@pharma.ethz.ch)


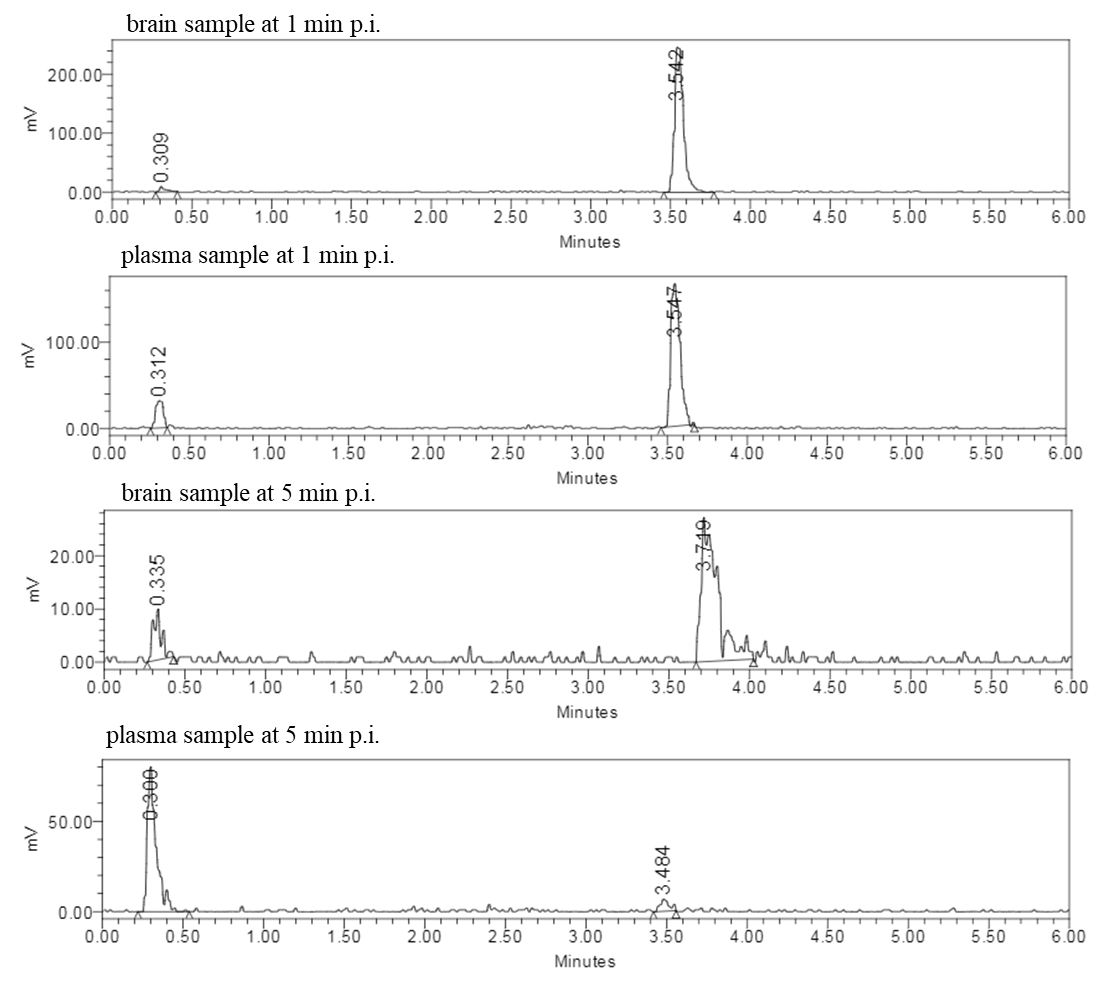


**Figure 1.** The representative UPLC chromatograms of (*R*)-[^11^C]YH132 from *ex vivo* radiometabolite studies in mice.


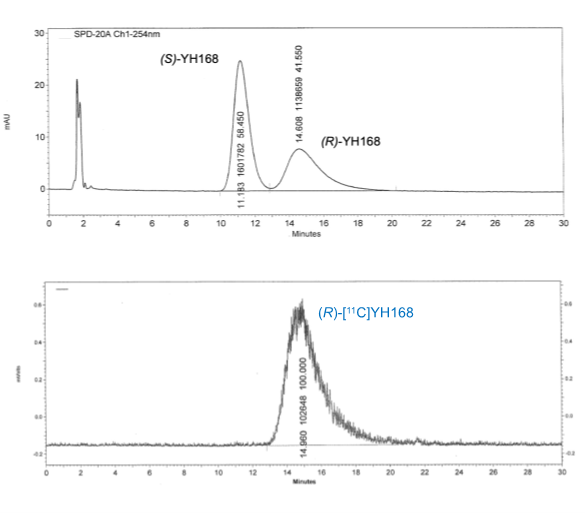


**Figure 2.** The representative HPLC chromatograms of (*R*)-[^11^C]YH168 using a chiral column.

**Figure 3.**  TACs of (*R*)-[^11^C]YH132 and (*R*)-[^11^C]YH168 in MAGL knockout and wild-type mouse brains.^*^The data was previously published by He *et al.* [1].


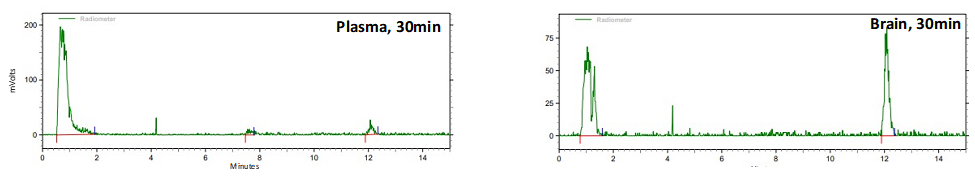


**Figure 4.** *Ex vivo* radiometabolite studies of (*R*)-[^11^C]YH168 at 30 min post-injection.


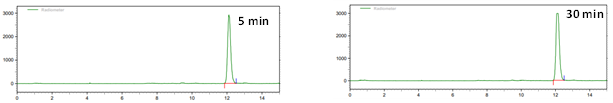


**Figure 5.** *In vitro* stability of (*R*)-[^11^C]YH168 in freshly prepared C57/Bl6 mouse brain homogenates after 5 and 30 min incubation at 37 °C.

**Scheme 1**. Synthetic procedure of (*R*)-YH168 and corresponding precursor. Reagents and conditions: a) triethylamine, tetrabutylammonium iodide, CH_3_CN/toluene, 50 °C, 4-iodoaniline, overnight; b) conc. hydrochloric acid, CH_3_CN, 50 °C, 4-5 h; c) sodium cyanoborohydride, acetic acid, anhydrous THF, r.t, furan-2-yl(piperazin-1-yl)methanone, overnight; d) 1,3-phenyldiboronic acid, potassium acetate, [1,1′-bis(diphenylphosphino)ferrocene]dichloropalladium(II), anhydrous DMF, 80 °C, 4 h; e) cesium carbonate, *tris*(dibenzylideneacetone) dipalladium(0), Sphos, anhydrous dioxane, *m-*tolylboronic acid, overnight; f) chiral supercritical fluid chromatography separation.

**Table 1**. Characterizations of (*R*)-YH132, (*R*)-YH168, (*R*)-[^11^C]YH132 and (*R*)-[^11^C]YH168.

| **Compound** | | **(*R*)-YH132** | **(*R*)-YH168** | **Radiotracer** | **(*R*)-[^11^C]YH132** | **(*R*)-[^11^C]YH168** |
| --- | --- | --- | --- | --- | --- | --- |
| *IC*_50_ (nM, mean ± SD) | Mouse MAGL | 4.1 ±1.7 | 3.8 ± 2.1 | Free fraction in mouse plasma (%, mean ± SD) | 23 ± 1 | 14.7 ± 0.2 |
|  | Cynomolgus monkey  MAGL | 6.2 ± 2.3 | 2.7 ± 1.3 |  |  |  |
|  | Human  MAGL | 5.4 ± 1.6 | 4.0 ± 1.3 |  |  |  |
| Liver microsome Cl_int_ (mouse, μL/min/mg protein) | | 189 | 388 | Percentage of intact structure 5 min post-injection (mouse brain sample, %) | 85 | 92 |
| cLog P from ChemDraw 22.0.0 | | 4.1 | 4.7 |  |  |  |
| PAMPA_P_EFF_ (cm/s*10^-6^) | | 4.0 | 15.8 | Percentage of intact structure 5 min post-injection (mouse plasma sample, %) | 8 | 34 |

**Reference**

1. He Y, Delparente A, Jie CVML, Keller C, Humm R, Heer D, et al. Preclinical Evaluation of the Reversible Monoacylglycerol Lipase PET Tracer (*R*)-[^11^C] YH132 : Application in Drug Development and Neurodegenerative Diseases. ChemBioChem. 2024;202300819. Available from: <https://doi.org/10.1002/cbic.202300819>
